# Evaluation of in vivo mutagenesis for assessing the health risk of air pollutants

**DOI:** 10.1186/s41021-016-0064-6

**Published:** 2017-04-01

**Authors:** Yasunobu Aoki

**Affiliations:** National Institute for Environmental Studies, Center for Health and Environmental Risk Research, 16-2 Onogawa, Tsukuba, Ibaraki 305-8506 Japan

**Keywords:** Air pollutant, Environmental mutagen, Genotoxic carcinogen, In situ exposure, Lung, Mutation hotspot, Polycyclic aromatic hydrocarbon, Risk assessment, Transgenic rodent gene mutation assay

## Abstract

Various kind of chemical substances, including man-made chemical products and unintended products, are emitted to ambient air. Some of these substances have been shown to be mutagenic and therefore to act as a carcinogen in humans. National pollutant inventories (e.g., Pollutant Release and Transfer Registration in Japan) have estimated release amounts of man-made chemical products, but a major concern is the release of suspended particulate matter containing potent mutagens, for example, polycyclic aromatic hydrocarbons and related compounds generated by the combustion of fossil fuel, which are not estimated by PRTR system. In situ exposure studies have revealed that DNA adducts in the lung, and possibly mutations in germline cells are induced in rodents by inhalation of ambient air, indicating that evaluating in vivo mutations is important for assessing environmental health risks. Transgenic rodent systems (Muta, Big Blue, and *gpt* delta) are good tools for analyzing in vivo mutations induced by a mixture of chemical substances present in the environment. Following inhalation of diesel exhaust (used as a model mixture), mutation frequency was increased in the lung of *gpt* delta mice and base substitutions were induced at specific guanine residues (mutation hotspots) on the target transgenes. Mutation hotspots induced by diesel exhaust were different from those induced by benzo[*a*]pyrene, a typical mutagen in ambient air, but nearly identical to those induced by 1,6-dinitropyrene contained in diesel exhaust. Comparison between mutation hotspots in the *TP53* (p53) gene in human lung cancer (data extracted from the IARC TP53 database) and mutations we identified in *gpt* delta mice showed that G to A transitions centered in CGT and CGG trinucleotides were mutation hotspots on both *TP53* genes in human lung cancers and *gpt* genes in transgenic mice that inhaled diesel exhaust. The carcinogenic potency (TD_50_ value) of genotoxic carcinogen was shown to be correlated with the in vivo mutagenicity (total dose per increased mutant frequency). These results suggest that the mutations identified in transgenic rodents can help identify environmental mutagens that cause cancer.

**Electronic supplementary material:**

The online version of this article (doi:10.1186/s41021-016-0064-6) contains supplementary material, which is available to authorized users.

## Background

Various chemical substances, including man-made products and unintended products, have been and continue to be emitted into the environment, such as ambient air, water, and soil; of these environmental media, ambient air is the major destination of pollutants. The Japanese national inventory for the release of chemicals to the environment (Pollutant Release and Transfer Registration, PRTR) [1] contains statistics of releases notified by businesses (point sources) and the releases outside notification (i.e., non-point sources, such as automobile and household emissions) estimated by the government; in the fiscal year 2014, about 90% of the total release of man-made chemicals from point sources was emitted into the air; even if the release from non-point sources was included, it is estimated that 80% of the total release was emitted into the air. PRTR also shows the release of IARC/WHO (International Agency for Research on Cancer/World Health Organization) Group 1 substances (categorized as human carcinogens), into the environment. For example, the release of benzene and 1,3-butadiene (1,3-BD) in fiscal year 2013 was estimated to be 7700 and 1500 t, respectively (including 7000 and 1300 t, respectively, emitted from non-point sources) [2]. These data indicate that measurable amounts of carcinogens are released to ambient air, although the annual average concentrations of these substances in ambient air were not over the environmental quality standard (3 μg/m^3^ for benzene) and the guideline value (2.5 μg/m^3^ for 1,3-BD) at any monitoring stations in urban areas in Japan in fiscal year 2013 [3].

While the release of man-made chemicals has been comprehensively estimated in industrial countries by governments and non-governmental organizations, registration of the amount of unintended products released to the environment is limited; for example, dioxins are the only unintentionally-produced by-product whose release has been registered in PRTR in Japan [2]. However, it is well-known that various kinds of unintentionally-produced by-products are generated by the combustion of fossil fuel, for example, in automobile engines (such as diesel engines) and in heating processes, and are emitted mainly to ambient air as components of suspended particulate matter (SPM). Some unintended products in ambient air are suspected to cause lung cancer and other respiratory disease, such as asthma [4–6].

The soot generated by combustion of coal was the earliest-discovered air-born carcinogen; in the 18^th^ century, it was discovered that heavy exposure to soot causes skin tumors in humans, and in the early 20^th^ century, benzo[*a*]pyrene (BaP), a polycyclic aromatic hydrocarbon (PAH) contained in soot, was identified as a carcinogen (for review, see [7]). In recent decades, some PAHs, including nitrated PAHs, contained in SPM have been shown to be genotoxic and have been categorized as IARC Group 1 (e.g., BaP) or IARC Group 2A/2B (suspected human carcinogens) [8]. For instance, the exhaust of diesel engines is classified as IARC Group 1 [9]. These facts indicate that mixtures of mutagenic and/or carcinogenic combustion products have been released into the air and inhaled by habitats in urban areas.

Epidemiological studies in the United States [10, 11] and Japan [12] show an association between the incidence of disease (lung cancer and respiratory disease) and long-term exposure to air pollution including SPM with a diameter below 2.5 μm (PM2.5). Recent investigations conclude that outdoor air pollution is classified as IARC Group 1 [13]. However, the cancer risk of air pollutant mixtures that contain mutagenic by-products of combustion has only been evaluated on the basis of limited epidemiological data. To improve the health risk assessment of whole air pollutants, it is necessary to conduct experimental animal studies to quantitatively evaluate how the exposure to a mixture of air pollutants induces DNA damage, such as DNA adducts, that can lead to in vivo mutagenesis and potentially carcinogenesis; furthermore, it is important to know whether such air pollutants could induce mutations in germline cells.

### Induction of DNA damage by inhalation of mutagens

The presence of mutagens in ambient air, especially in SPM, has been surveyed in various countries by using in vitro bioassay systems, such as the Ames test [14, 15]. Studies in Japan have shown that mutagens are ubiquitously present in air-borne particles collected in large cities [16]. Matsumoto et al. [17] reported that the contents of the PM2.5 fraction collected at an intersection with heavy traffic in Tokyo exerted higher mutagenicity than did larger air-borne particles from the same location. Watanabe et al. [18] showed that the soil in the Kyoto area contained mutagenic compounds that might be deposits from air, and identified the major mutagens as nitrated PAHs such as 3,6-dinitrobenzo[*e*]pyrene. The identification of various mutagens in ambient air indicates that people inhale a mixture of various mutagens, rather than a single mutagen.

Inhalation of a mixture of mutagens has been suspected to induce DNA damage resulting in carcinogenesis in target organs and, in some cases, mutagenesis in the germ cells. Although DNA adduct formation, micronucleus induction, and DNA strand breaks in surrogate tissues, for example white blood cells, have been analyzed as biomarkers for assessing the genotoxicity of tobacco smoke [19, 20], the total mutagenicity of the environmental mixture in ambient air remains to be clarified. Formation of DNA adducts has been shown to be elevated in the white blood cells of individuals heavily exposed to air pollutants [21]; however, the amount of DNA adducts induced in target tissues, especially lung, by air-borne chemicals needs to be analyzed to assess the mutagenicity of the whole environmental mixture. Since analysis of DNA adducts in lung tissue cannot be conducted for human populations, studying the exposure of experimental animals, such as rodents, to ambient air (in situ exposure), is a limited but potentially effective method for addressing the issue of how a whole mixture of air pollutants is mutagenic [22].

### In situ exposure of experimental animals to ambient air

We conducted an in situ exposure study for a 60-week period from 1996 to 1997 in which rats were maintained in polluted air containing SPM with a diameter of <11 μm at an average monthly concentration of 76.5 μg/m^3^ (range 50.4–108.3 μg/m^3^). The rats were housed in a small animal facility located beside an intersection with heavy traffic in the Tokyo metropolitan area, and air was circulated from the outside environment [23]. DNA adduct analysis revealed that the levels of DNA adducts, some of which were probably PAH–DNA adducts, were elevated in lungs, nasal mucosa, and livers of rats after exposure to urban air for 4 weeks, with the levels decreasing gradually during prolonged exposure over 12 weeks; these results indicate that urban air exerts genotoxic effects not only directly in respiratory organs, such as lung and nasal mucosa, but also indirectly in tissues remote from respiratory organs, such as liver. An in situ exposure study in São Paulo showed induction of micronuclei in peripheral erythrocytes of mice following exposure to urban air for 90 days [24]. In an earlier in situ exposure study, coke oven emissions containing 892 ng/m^3^ PAHs, as a model mixture of air pollution, were shown to induce DNA adduct formation in the lungs and non-respiratory tissues (heart, liver, and white blood cells) of rats [25]. These results suggest that air pollution in urban areas causes somatic mutations.

Analysis of mutagenesis not only in somatic cells but also in germ cells has been a major issue in environmental mutagen research. In situ exposure to ambient air in a polluted area near a steel mill in Canada in 1999 showed that the frequency of inheritable mutations at expanded simple tandem repeat (ESTR) loci was significantly higher in mice exposed to polluted ambient air for 10 weeks compared to those in a non-polluted area [26]; ESTR mutations are detected as a change in the number of tandem repeats in the ESTR loci (e.g., Ms6-hm). Interestingly, the elevation of heritable mutations at the ESTR loci was originally observed in a herring gull (*Larus argentatus*) population nesting in a polluted area near steel mills [27, 28]. Somer et al. [29, 30] and Yauk et al. [31] demonstrated that the frequency of ESTR mutations in mice exposed to polluted air near steel mills and a highway was 1.6 times higher than that of mice exposed to air that was SPM depleted by high-efficiency particulate arrestance (HEPA)-filtration at the same location, indicating that SPM can potentially increase the mutation frequency at ESTR loci. DNA strand breaks in sperm and DNA-adduct formation in lung were also found to be elevated in mice exposed to polluted air containing total SPM (mean 93.8 ± 17.0 μg/m^3^) or PAHs (mean 8.3 ± 1.7 ng/m^3^), for 3 or 10 weeks, respectively [31]. These increases in heritable mutations at ESTR loci suggest that exposure to air-borne particulates containing mutagens can cause germ cell mutations. An elevated frequency of mutations at ESTR loci was also observed in mouse sperm following exposure of the mice to tobacco smoke (mainstream tobacco smoke [32] or sidestream tobacco smoke [33]). Irradiation studies showed that the average doubling dose of ESTR mutations following low linear energy transfer (LET) irradiation of spermatogonia or stem cells was 0.62–0.69 Gy [34]. However, the mechanism for inducing ESTR mutations is still unknown, and further studies are required to resolve the biological significance of ESTR mutations [34].

### Transgenic rodents as a tool for analyzing in vivo mutagenesis by air pollutants

The observations in the above in situ exposure studies raise the question of whether DNA damage induced by exposure to air pollutants becomes fixed as in vivo mutations in the target tissues. To answer this question, transgenic rodents (Muta, Big Blue, and *gpt* delta) are useful, because they allow analysis of in vivo mutations induced by environmental mutagens.

In the Muta, Big Blue, and *gpt* delta systems, a target gene for detecting mutations (*lacZ* encoding *E. coli* β-galactosidase, *lacI* encoding the *E. coli* lactose operon repressor, or *gpt* encoding *E. coli* guanine phosphoribosyltransferase, respectively) carried on a λ phage shuttle vector is integrated into the genomic DNA (for review, see [35]). Transgenic mice, harboring genome-integrated DNA plasmids containing the *lacZ* gene were also established [36, 37]. After Muta, Big Blue, and *gpt* delta rodents are exposed to mutagen, the shuttle vector is rescued from the genomic DNA to host *E. coli*, and (1) the mutated target gene is detected by phenotypic transformation of the host *E. coli*, that is the appearance of β-galactosidase-null plaques, β-galactosidase-expressing plaques, and 6-thioguanine-resistant colonies, respectively, (2) the *cII* gene on the λ phage shuttle vector can also be used as the target gene (for review, see [35]), and (3) the *gpt* delta system can be applied to detect large deletions on genomic DNA.

Transgenic rodents have been used for the analysis of in vivo mutagenicity of carcinogenic air-borne chemicals such as 1,3-BD, ethylene oxide, and PAHs. For example, inhalation of 1,3-BD at a concentration of 625 ppm by Muta mice for 5 days accelerated in vivo mutagenesis in lung but not in bone marrow or liver [38]. When Big Blue mice were exposed to 1,3-BD at the same concentration and for the same time period as that used for Muta mice above, the results demonstrated that 1,3-BD is mutagenic in bone marrow [39]. Recio et al. [39] proposed that the difference in in vivo mutagenicity of 1,3-BD in bone marrow between Muta mice and Big Blue mice might be due to differences in 1,3-BD bioactivation between the host strains (CD2F1 and B6C3F1, respectively). Following 2-year inhalation exposure to 1,3-BD, an increased incidence of neoplasms in the hematopoietic system was shown in B6C3F1 [40]. Furthermore, when Big Blue mice were exposed to 1,3-BD at a concentration of 1250 ppm for 4 weeks, the frequency of mutations at A:T pairs was significantly increased in bone marrow [41], and the frequency of G to A transitions was significantly increased at non-CpG sites in spleen [42, 43]. Formation of persistent DNA adducts derived from 1,3-BD on adenine residues [44] may contribute to the increased frequency of mutation at A:T pairs. Elevation of in vivo mutagenesis in lung by ethylene oxide was also demonstrated by an inhalation study using Big Blue mice [45, 46]. The relative potencies of in vivo mutagenicity were compared among PAHs administered by intraperitoneal (ip) injection to Big Blue mice [47] or orally to Muta mice [48]. The results indicated that in vivo mutagenicity in lung was as high for dibenzo[*a,l*]pyrene as for BaP; however, whereas G to T transversions and G to A transitions were the major base substitutions induced in lung by dibenzo[*a,l*]pyrene, G to T transversions were the sole major base substitution induced in lung by BaP [47]. Our group’s intratracheal administration studies showed that the mutant frequency in vivo of 1,6-DNP [49] was about 20 times higher than that of BaP [50]. These observations indicate that transgenic rodent assay systems are a useful tool for analyzing in vivo mutations induced in lung and other respiratory organs by environmental chemicals inhaled from ambient air.

### Analysis of in vivo mutagenesis induced by diesel exhaust as a model mixture of air pollutants

By using various transgenic rodents, our group examined the in vivo mutagenicity of diesel exhaust emitted from an engine. Diesel exhaust is an appropriate model of air pollution in urban areas, because it is the major source of genotoxic carcinogens, such as PAHs, as mentioned above. We showed that inhalation of diesel exhaust, at an SPM concentration of 6 mg/m^3^ for 28 days, by Big Blue rats increased the mutant frequency, and elevated the level of DNA adducts, in lung [51]. In contrast, feeding of standard reference material of diesel particles at a concentration 80 mg/kg body weight for 21 days increased the level of DNA damage in colon and liver [52] and lung [53] of Big Blue rat, but did not elevate in vivo mutagenicity in these organs.

Our inhalation study using *gpt* delta mice also demonstrated that the frequency of in vivo mutation increased in lung depending on the duration of exposure to diesel exhaust at an SPM concentration of 3 mg/m^3^ [54]. The mutant frequency was elevated to 2.11 ± 0.08 × 10^−5^ in exposed mice compared with 0.82 ± 0.07 × 10^−5^ in control mice, after 24-weeks exposure. However, inhalation of standard reference material of diesel particles for 90 min at a concentration of 80 mg/m^3^ on a single day or 4 consecutive days did not elevate the in vivo mutagenicity in the lungs of Muta mice [55]. Our studies using transgenic rodents demonstrate that diesel exhaust emitted from an engine induces in vivo mutagenesis in lung, whereas induction of micronuclei in reticulocytes was not observed in mice and rats by exposure of diesel exhaust at the low concentration (about 30 μg/m^3^) for 3 months [56, 57].

Sequence analysis of mutated *gpt* genes rescued from the diesel exhaust–exposed lungs of *gpt* delta mice revealed that the most frequent type of base substitution on *gpt* gene induced by exposure to diesel exhaust was G to A transitions [58], and the next most common was the G to T transversions. Mutation hotspots (mutation loci identified from three or more mice) were present at nucleotide numbers 64, 110, and 115 for G to A transitions and 185, 401, 402, 406, and 418 for G to A transitions and G to T transversions; this is recognized as a molecular signature for mutation induced by oxidative stress [59] or exposure to BaP in lung [50]. Since nucleotide numbers 64 and 110 were sites of mutation in non-exposed mice, the components in diesel exhaust possibly enhanced the frequency of spontaneously-induced mutations, but mutations at nucleotide numbers 185, 401, 402, 406, and 418 may be a unique signature for exposure to diesel exhaust. Interestingly, these hotspots induced by diesel exhaust were different from those induced by intratracheally-administered BaP, that is G to T transversions at nucleotide numbers 125, 140, 143, and 413 [50], but similar to those induced by intratracheally administered 1,6-DNP [49], suggesting that components other than BaP in diesel exhaust were the major inducers of in vivo mutagenesis in lung. Hotspots of mutation in the livers of phenacetin-dosed *gpt* delta rats, that is nucleotide numbers 26 and 416 [56], are different from those induced by exposure to diesel, 1,6-DNP, or BaP. Therefore, hotspots of mutation in target genes integrated into the genomic DNA of rodents are potentially specific landmarks for identifying a type of exposed mutagen.

### Germline mutations induced by mutagens contained in diesel exhaust

Following inhalation of diesel exhaust to *gpt* delta mice, we observed an increase in the mutant frequency in testis, but not in liver, suggesting that diesel exhaust or mutagens in diesel exhaust might induce mutations in male germline cells such as sperm [58]. However, this observation contrasted with in vivo mutagenicity of a heterocyclic amine, 2-Amino-1-methyl-6-phenylimidazo[4,5-b]pyridine (commonly known as PhIP), which induced mutations in liver but not in testis [60]. Any mutagens in diesel exhaust may be specifically distributed to the testis and induce mutations in this organ. Abnormal morphology of sperm is reported to be correlated to the level of air pollution in the human population of the Czech Republic [61], but it is yet to be revealed whether air pollution causes germline mutations.

Transgenic rodents are also a good model for evaluating induction of germline mutations. Administration of a potent mutagenic alkylating agent, ethylnitrosourea or isopropyl methanesulfonate, to Muta mice by a single ip injection induced mutations in the *lacZ* target gene, and genomic DNA rescued from seminiferous tubule germ cells after a period of mutation expression for 52 days, showed a significant increase in the mutant frequency in germline cells [62]. When inherited germline mutations induced by ethylnitrosourea were examined using *gpt* delta mice, the frequency of inherited mutations in the offspring of ethylnitrosourea-treated mice was 17-fold higher than that of the offspring of control mice [63]. A longer expression period was required to detect mutations in germline cells than somatic cells, because it takes a prolonged period to fix mutagen-induced DNA lesions in stem cells of germline as a mutation in differentiated germline cells [64].

Regarding environmental mutagens, germ cell mutations induced by BaP have also been examined in transgenic rodents. After transgenic mice bearing pUR288 *lacZ* were administered BaP by gavage at a dose of 13 mg/kg body weight 3 times per week for 6 weeks, DNA adducts were induced in testis; however, the mutant frequency increased in sperm but not in testis [65]. In contrast, an increased mutant frequency in sperm was not observed in mice homozygous deficient for the *Xpc* gene, which plays a role in the removal of bulky-DNA adducts [65]. Formation of BaP–DNA adducts in stem cell spermatogonia was shown to give rise to de novo mutations in the sperm [66, 67], and exposure to BaP in earlier life (5-days-old) induced elevated mutant frequency in spermatogenic cells more effectively than exposure to BaP in the adult stage [68]. A single-molecule PCR method was established to measure induction of mutations at ESTR loci [69]. Using this method, administration of BaP by gavage at a dose of 100 mg/kg body weight for 28 days was shown to induce ESTR mutations in the sperm of Muta mice [70].

Male germline mutations at ESTR loci were also enhanced in the offspring of female mice who inhaled standard reference material of diesel particles [71] or were subjected to irradiation by X-ray [72]. These observations suggest that BaP and other mutagens in diesel exhaust could be germ cell mutagens. An interesting comparative study has reported that one adduct formation of BaP has approximately the same mutagenicity as 10^−4^ Gy ɤ-irradiation [73]. Further studies, similar to those for ethylnitrosourea [74], are required to quantitatively evaluate germ cell mutagenesis induced by low-dose sub-chronic exposure to BaP.

### Mutation on *Ras* proto-oncogenes and *TP53* (tumor suppressor gene) in somatic cells by environmental mutagens

Induction of somatic mutation(s) at specific sequences on proto-oncogenes and/or tumor suppressor genes is a key process in carcinogenesis. To reveal how mutation at these specific sequences is induced by environmental mutagens is an important issue for understanding the mechanism of mutagenesis and carcinogenesis induced by environment mutagens. Furthermore, mutations on the unique sequences are candidate molecular signatures for monitoring the exposure of mutagens.

Observations of mutations at codons 12, 14, or 61, of *Ras* genes in human cancer have been well-documented [75]. In chemical carcinogenesis studies, the *Hras* gene was mutated at codon 61 in mouse hepatomas induced by exposure to *N*-hydroxy-2-acetylaminofluorene, vinyl carbamate, or 1′-hydroxy-2′,3′-dehydroestragole [76], and analysis of DNA adduct formation revealed the binding of benzo[*a*]pyrene diol epoxide (BPDE, reactive intermediate of BaP) to guanine or adenine in codons 12 and 14 or of *Kras* gene in cultures of normal human bronchial epithelial cells treated with BPDE [77].

In vivo mutagenesis of the *Kras* gene has been analyzed by allele-specific competitive blocker PCR (ACB-PCR). After Big Blue rats were treated with *N*-hydroxy-2-acetylaminofluorene, the frequencies of GGT to GTT and GGT to GAT substitutions at *Kras* codon 12 in liver were 3.3 and 6.4 times, respectively, those in the control, while transgenic rodent assay showed that G to T transversion was the major base substitution induced by *N*-hydroxy-2-acetylaminofluorene [78]. In contrast, treatment of Big Blue rats with aristolochic acid significantly increased the frequency of GAA to GTA substitutions at *Kras* codon 61 in liver and kidney, but did not induce GGT to GAT substitution at *Kras* codon 12 [79]. The frequencies of GGT to GTT and GGT to GAT substitution at *Kras* codon 12 were elevated by inhalation of ethylene oxide at 100 ppm and 50–200 ppm, respectively, for 4 weeks, but this effect was not observed following inhalation for 8 weeks [80]; the authors speculated that negative selection against cells carrying *Kras* mutations occurred at the high cumulative dose of ethylene oxide. The results suggest that environmental mutagens induce mutations site-specifically on the *Kras* gene.

Mutations in the *TP53* (p53) tumor suppressor gene are frequently observed in human cancer [81, 82]. The IARC TP53 database [83] compiles data on *TP53* mutations detected in human cancer, and provides useful information for understanding the mechanism of carcinogenesis. Analysis of this database reveals that the *TP53* gene is mutated frequently at several specific codons in cancer. For example, in BPDE-treated HeLa cells and bronchial epithelial cells, BPDE–DNA adducts frequently form at commonly mutated codons in the *TP53* gene (codons 157, 248, and 273) [84], suggesting that a unique mutation spectrum was induced by each mutagen on the *TP53* gene. To compare the *TP53* mutation spectra produced by various environmental mutagens, human p53 knock-in (Hupki) mouse lines were established and an in vitro assay for detecting mutations induced on the human *TP53* gene was developed [85]. In the Hupki mouse genome, the DNA-binding domain of the mouse *Trp53* (Tp53) gene has been replaced with the normal human *TP53* gene by using gene-targeting technology. For the in vitro assay, cultures of Hupki mouse–derived embryonic fibroblast (HUF) cells were treated with chemical substances; immortalized cells were obtained following several passages of treated cells; and the human*TP53* gene in the immortalized cells was subjected to sequencing.

Treatment of HUF cells with BaP frequently induced mutations including G to T transversions (a landmark mutation of BaP exposure) on codons 157, 158, and 273 of the *TP53* gene; these correspond to positions frequently mutated in human lung cancer [86, 87]. A potent mutagen present in diesel exhaust particles, 3-nitrobenzanthrone, induces G to T transversions in HUF cells [88]. In HUF cells treated with aristolochic acid, an A to T transversion is induced in codon 139, which is registered as a frequently mutated site in the IARC p53 mutation database [89]. Taken together, these observations indicate that HUF cells are potentially useful for identifying mutagen-specific mutation sites on the *TP53* gene, and thus can be used to reveal the mechanisms by which environmental mutagens cause carcinogenesis.

### *TP53* gene mutation in lung cancer and molecular signature induced by inhalation of environmental mutagens

Inhalation of mutagens is recognized to cause lung cancer, and air pollutants and tobacco smoke are suspected to be major causes of in vivo mutagenesis of proto-oncogenes and tumor suppressor genes in lung. Among proto-oncogenes and tumor-suppressor genes, *TP53* is frequently mutated gene in lung cancer; about 40% of all lung cancer cases compiled in the IARC TP53 database [83] carry a mutated *TP53* gene. A unique characteristic of *TP53* mutation in lung cancer is a high rate of occurrence of G to T transversions; this rate is comparable to that of G to A transitions, which are common mutations in the *TP53* gene in all types of cancer, including lung [81, 90]. The frequently mutated codons (hotspots) on the *TP53* gene in lung cancer are codons 157, 158, 175, 245, 248, 249, and 273 [91].

G to T transversion is a base substitution induced not only by administration of BaP via formation of BPDE-DNA adducts [50, 92] but also by generation of reactive oxygen species via formation of 8-oxo-deoxyguanine [93, 94]. Because the lung is an organ directly in contact with air, it is reasonable to expect that G to T transversions that are induced by exogenous agents such as PAHs or oxygen would occur frequently in lung cancer. Mutation spectrum analysis has shown that the rate of G to T transversions in the lung cancer of smokers (about 30%) is higher than that of non-smokers (10%–15%) [90, 91, 95]. However, experimental inhalation of environmental tobacco smoke to Big Blue mice showed that the most common mutation induced on the *cII* gene was G to A transition and the next was G to T transversion [96].

I analyzed the IARC TP53 database to reveal the mutation spectrum at the level of nucleotide sequence of the *TP53* gene in lung cancer, and potentially identify agent(s) contributing to mutagenesis of the *TP53* gene. Table 1 summarizes my analysis of the base substitutions in frequently mutated codons in the *TP53* gene in lung cancer [91]. It is well-known that mutations are mainly induced at CpG sites on the *TP53* gene in human cancer [81]. As shown in Table 1, G to T transversions were induced in lung cancer on 5 guanine residues centered in CGN triplets at nucleotide #12457 of codon 157 (CGT to CTT), #12461 of codon 158 (CGC to CTC), #13370 of codon 245 (CGG to CTG), #13380 of codon 248 (CGG to CTG), and #13799 of codon 273 (CGT to CTT). The triplets (CGC, CGT, and CGG), in which G to T transversions were induced in the *TP53* gene, were identical to those containing the BaP-induced mutation hotspots (nucleotide numbers 125, 140, 143, and 413 on the *gpt* gene) in the lungs of *gpt* delta mice [50]. These observations confirm the speculation that G to T transversions on mutated *TP53* genes in lung cancer may be induced by BaP and other carcinogenic PAHs contained in tobacco smoke [90, 91, 97].

**Table 1:**
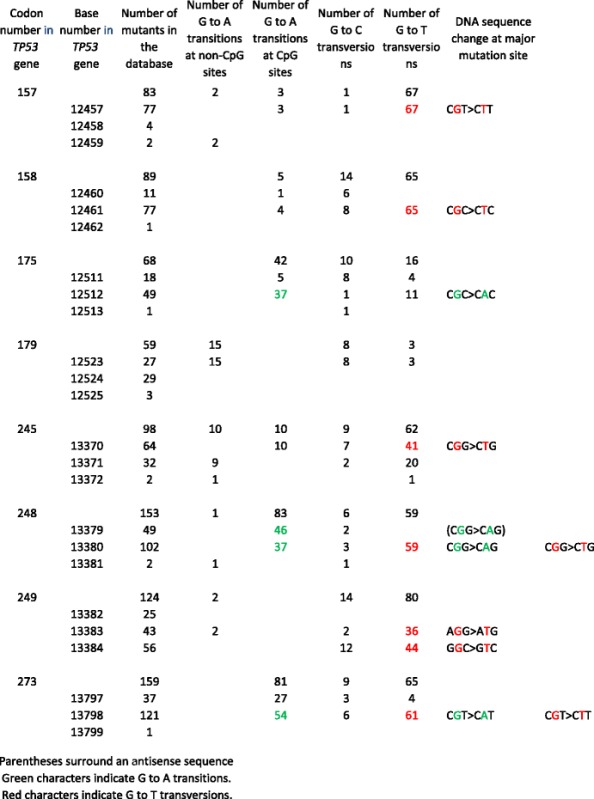
ᅟ

G to A transitions were also frequently induced in lung cancer. G to A transitions were induced at 4 guanine residues, that is, at nucleotide #12512 of codon 175 (CGC to CAC), #13380 and #13381 of codon 248 (CGG to CAG), and #13799 of codon 273 (CGT to CAT). Among these triplets, guanine residues centered in CGT and CGG were also mutation hotspots for G to A transitions induced by inhalation of diesel exhaust in the lungs of *gpt* delta mice (nucleotide numbers 64, 110, and 115 of the *gpt* gene) [54]. Again, these findings indicate that air pollutants emitted from diesel engines and other fossil fuel combustion processes may contribute, at least partly, to mutagenesis of the *TP53* gene, but the possibility that spontaneous mutations were enhanced on frequently mutated codons on *TP53* gene cannot be ruled out, because G to A transitions occur frequently as spontaneous mutations. Nevertheless, it is clear that comparison between the mutation spectra of proto-oncogenes and tumor suppressor genes in lung cancers and those of in vivo mutations in transgenic rodent assays can provide clues to the identify of environmental mutagens that cause cancer.

### Correlation between in vivo mutagenicity and carcinogenicity

As mentioned above, transgenic rodents are a good tool for evaluating the potential of environmental chemicals to induce mutations in vivo. However, more investigation is required to reveal the relationship between the potencies of in vivo mutagenicity and carcinogenicity. Suzuki [98] reported that TD_50_ (value of chronic dose-rate [mg/kg body weight/day] that would induce tumors in half the test animals at the end of a standard lifespan [99]) was associated with fold-increase in mutant frequency in transgenic rodents per total dose administered to the animals. Recently, Ono and Honma [100] presented the possibility that BMDL_10_ (the lower 95% confidence bound on the benchmark dose corresponding to 10% specific change) of carcinogenicity can be predicted from the BMDL_10_ of mutagenicity in the transgenic rodents. Therefore, it is important to precisely analyze the relationship between in vivo mutagenicity in the transgenic rodent assay and the carcinogenicity of various chemicals.

For this purpose, I extracted dose–response data for carcinogenicity from the Carcinogenic Potency Database (CPDB) [99], which contains the dose–response data for 1547 chemicals; and I obtained data on in vivo mutagenicity of 163 chemicals in transgenic rodent assays from the published literature [35]. At the Annual Meeting of Japanese Environmental Mutagen Society in 2009 [101], based on preliminary data, I put forward the hypothesis that TD_50_ values of genotoxic carcinogens correlate to the values of in vivo mutagenic potency if compared for the same target organ and administration route. Because the TD_50_ value in CPDB is the harmonic mean calculated from the TD_50_ value of the most potent target site, in the current study I obtained TD_50_ values of genotoxic carcinogens in liver and lung for each route of administration (inhalation, oral gavage or diet, or ip injection) by calculating harmonic means of the values cited in CPDB except benzene (see Additional file 1). I defined in vivo mutagenic potency as the harmonic mean of the total dose of agents administered to an animal (total dose) divided by the induced mutant frequency (the mutant frequency of the treatment group minus the mutant frequency of the control group) × 10^5^ (IMF); the values for total dose and IMF used for this calculation were extracted from reference [35].

The criteria of dose–response data used for calculation of TD_50_ in mouse liver and lung were as follows: 1) dose–response data with two or more doses were used, and if this was not available, one-dose data were used; 2) if more than one tumor type was induced in the organ, data from the various tumor types was combined for the calculation; 3) if dose–response data with two or more doses were used, the dose–response curve that was consistent with linearity (marked as ‘*’ in CPDB) was used for the calculation; 4) the data in CPDB were evaluated as showing positive carcinogenicity (marked as c or + in the database); 5) the number of animals per group was over ten, 6) the incidence of tumors in the control and the elevation of incidence in treatment groups was below 40% and over 10%, respectively; and 7) the significance of the correlation of dose–response was *P* < 0.10. For calculating in vivo mutagenic potencies, total doses obtained by multiple-time dosing cited in reference [35] were used, except for total dose for inhalation, which was re-calculated as indicated in Additional file 1. The calculated values of the harmonic means of TD_50_ and total dose/IMF (T/I) of each chemical and the data for calculating these values are listed in Table 2 and Additional file 1, respectively.

**Table 2:** Harmonic means of TD_50_ values and total dose/IMF (T/I; potency of in vivo mutagenicity) values of genotoxic carcinogens in mouse liver and lung

Tissues	Compounds (administration method)	Harmonic mean of TD_50_ (mg/kg/day)	Harmonic mean of T/I (mg/kg)
Liver	2-acetylaminofluorene (diet)	7.81	231
	4-aminodiphenyl (diet)	1.58	15.3
	4-chloro-o-phenylenediamine (diet)	1341	7623
	2,4-diaminotoluene (diet)	26.7	1688
	dichloroacetic acid (diet)	155	66867
	MeIQ (diet)	27.1	272
	MeIQx (diet)	24.3	104
	*N*-nitrosodimethylamine (gavage)	0.253	0.69
	urethane (diet)	39	427
Lung	benzene (inhalation)	175	4937
	1,3-butadiene (inhalation)	41.8	532
	cyclophosphamide (ip injection)	5.92	16.6
	ethylene oxide (inhalation)	63.4	897
	urethane (diet)	28.3	303

The data for calculating these values are listed in Additional file 1

**Fig. 1:**
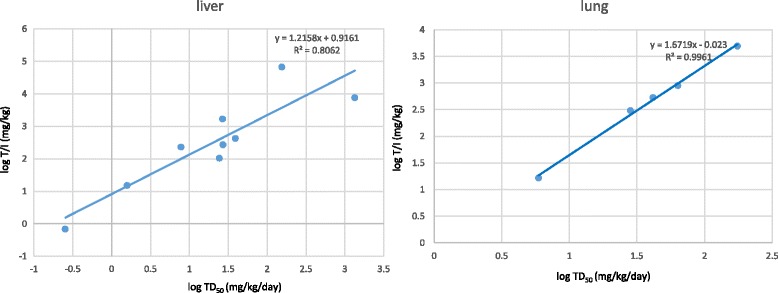
Plot of log [harmonic mean of TD_50_] (log TD_50_) vs log [harmonic mean of T/I] (log T/I) calculated by a statistical package in Excel 2010

I analyzed whether the harmonic mean values of TD_50_ of mouse liver or lung correlated to T/I values (in vivo mutagenic potencies). As shown in Fig. 1, log [harmonic mean of TD_50_] (hereafter, log TD_50_) was linearly related to log [harmonic mean of T/I] (hereafter, log T/I), suggesting that carcinogenicity of genotoxic carcinogens could be predicted from the target and route-matched in vivo mutagenicity. This linear correlation between log TD_50_ and log T/I was an unexpected finding, since the carcinogenicity of a genotoxic substance can be affected by the substance’s tumor promoting activity. Among various in vitro assay systems for predicting carcinogenicity, the Bhas assay, which uses a clone of BALB/c 3 T3 cells transfected with the v-Ha-*ras* gene, is an established system for evaluating the tumor promoter activity. The tumor promoter activity of several substances listed in Table 2 have previously been tested by Bhas assay; 2,4-diaminotoluene and urethane (ethyl carbamate) were evaluated to be negative, but 2-acetylaminofluorene was positive [102], indicating that, even if a genotoxic carcinogen possesses tumor promoter activity, the plots of carcinogenicity (log TD_50_) vs. in vivo mutagenicity (log T/I) show linearity. This observation suggests that the carcinogenicity of chemical substances that exert in vivo mutagenicity in the transgenic mouse system could be mainly driven by the mutagenicity (tumor initiator activity), at least, in liver. However, the mechanistic basis of this linear relationship remains to be further studied.

As a case study, the TD_50_ value of diesel exhaust was estimated from the harmonic mean of the T/I values (i.e., 105) in our study [58] (see Additional file 2). In a previous study, Ichinose et al. [103] demonstrated that the incidence of tumor in mouse lung was significantly elevated 3.1-fold by intratracheal administration of diesel exhaust particle at the dose of 0.1 mg/animal, repeatedly 10 times at weekly intervals, comparing with the vehicle control. Using the formula of the relationship between the carcinogenic potency and in vivo mutagenicity (Fig. 1), I estimate the TD_50_ of diesel exhaust to be 17 mg/kg/day (0.51 mg/animal/day, if body weight is 0.03 kg).

## Conclusions

By using transgenic rodent assay systems, one can detect mutations induced on genomic DNA at the level of alterations in the nucleotide sequence. These assay systems are a good tool for evaluating in vivo mutagenicity induced by inhalation of air pollutants, especially a whole mixture of air pollutants. Merits of these assay systems are that they enable 1) analysis of the mutation spectrum (the types of mutation, e.g., transition, transversion, or deletion, and their positions on the genome), and 2) quantitative measurement of the potency of in vivo mutagenicity in somatic cells and germ cells.

I therefore propose that 1) comparisons between the nucleotide sequences of mutation hotspots induced in somatic cells by exposure to mutagens and the frequently mutated nucleotide sequences on proto-oncogenes and tumor suppressor genes may provide information about the identify of mutagen(s) causing cancer; and 2) a linear relationship exists between log TD_50_ (carcinogenicity) and log T/I (in vivo mutagenicity), suggesting that carcinogenicity can be predicted from in vivo mutagenicity in the target organ of cancer. Data suggesting that air pollutants induce mutations on germ cells, as well as somatic cells, have been presented; however, further studies are required to assess the potency of mutation in germ cells from in vivo mutagenicity data. Recently, a high-throughput method for analyzing the mutation spectrum of target genes has been developed by using a next-generation sequencer [104, 105]. Novel technologies will strength a potential of transgenic rodent assay systems for assessing the carcinogenic risk of environmental mutagens.

A﻿ part of this review was presented in a lecture of JEMS Award 2016 in the Annual Meeting of Japanese Environmental Mutagen Society in 2016.

## Additional files

Additional file 1: The data for calculating TD_50_ and T/I. Parentheses: route of administration. Pathology: ade, adenoma; hpa, hepatoadenoma; hpc, hepatocarcinoma; hpt, hepatoma; mal, malignant tumor; mix, more than one tumor type; MXA, more than one tumor type, combined by National Cancer Institute/National Toxicology Program [99]. Transgenic Strain: BB, Big Blue mouse; Muta, Muta mouse; gpt, *gpt* delta mouse; Lac Z plasmid, transgenic mouse harboring plasmids containing the *lacZ* gene [36, 37].
**#TD**_**50**_ **for benzene calculated from the data** [106] **by the procedure of CPBD**. 300 ppm × 1/3 (1 week exposure followed by 2 weeks non-exposure) = 100 ppm. Incidence of lung adenoma in the exposed group and non-exposed group: 26% (15/54) and 7% (3/46), respectively. Concentration of benzene corresponding to 50% increased incidence (incidence of lung adenoma, 54%) = 250 ppm. 250 ppm × 78.11 (molecular weight)/24.45 L (standard volume) = 0.8 mg/L.
0.8 (mg/L) × 0.03 L/min (inhalation volume) × 60 (min) x 6 h/day (hours of inhalation per day/0.03 kg (body weight) = 288 mg/kg/day. Duration of exposure: 81 weeks. Life time of mouse: 104 weeks. **TD**_**50**_ **= 288 × (81 weeks/104 weeks)** ^**2**^ **= 175 mg/kg/day**.
The total doses were re-calculated at the conditions in the references cited in [35].
*benzene. 300 ppm × 78.11 (molecular weight)/24.45 (L, standard volume) = 0.96 mg/L.
0.96 (mg/L) × 0.03 L/min (inhalation volume) × 60 (min) x 6 h/day (hours of inhalation per day)/0.03 kg (body weight) × 5 days (inhalation days per a week) × 12 weeks (duration of inhalation) = **20,736 mg/kg**.
**1,3-butadiene. 625 ppm × 54.09/24.45 = 1.38 mg/L. 1.38 × 0.03 × 60 × 6/0.03 × 5 days (duration of inhalation) = **2,500 mg/kg**.
***ethylene oxide. 200 ppm × 44.05/24.45 = 0.36 mg/L. 0.36 × 0.03 × 60 x 6/0.03 × 5 × 4 = **2,600 mg/kg**. (XLSX 19 kb)

Additional file 2: Estimation of T/I for diesel exhaust. T/I of diesel exhaust was estimated based on our data [53]. (DOCX 13 kb)

## Abbreviations

1,3-BD: 1,3-butadiene
ACB-PCR: Allele-specific competitive blocker PCR
BaP: Benzo[*a*]pyrene
BPDE: Benzo[*a*]pyrene diol epoxide
CPDB: Carcinogenic potency database
ESTR: Expanded simple tandem repeat
HUF: Hupki mouse–derived embryonic fibroblast
Hupki: human p53 knock-in
IMF: Induced mutant frequency
ip: Intraperitoneal
PAH: polycyclic aromatic hydrocarbon
SPM: Suspended particulate matter
T/I: Total dose/IMF
TD_50_: Value of chronic dose-rate [mg/kg body weight/day] that would induce tumors in half the test animals at the end of a standard lifespan
