# Supplementary material for: Evaluation of in vivo mutagenesis for assessing the health risk of air pollutants
Source: Genes Environ. 2017 Apr 1;39:16. doi: 10.1186/s41021-016-0064-6 (PMC5376282; doi:10.1186/s41021-016-0064-6)
Supplement: Supplementary file 2 — Estimation of T/I for diesel exhaust. T/I of diesel exhaust was estimated based on our data [53]. (DOCX 13 kb) [file 41021_2016_64_MOESM2_ESM.docx]

**Supplemental Material 2**

Estimation of T/I for diesel exhaust.

Total dose 3 mg/m^3^ for 4 weeks;

0.003 (mg/L) × 0.03 L/min (inhalation volume) × 60 (min) × 12 h/day (hours of inhalation per day)/0.03 kg (body weight) × 7 days (inhalation days per week) × 4 weeks (duration of inhalation) = 60.5 mg/kg

IMF: 0.41 = 1.06 (exposed) – 0.61 (control))

T/I = 60.5/0.41 = 148

Total dose 1 mg/m^3^ for 12 weeks;

0.001 (mg/L) × 0.03 × 60 × 12/0.03 × 7 × 12 = 60.5 mg/kg

IMF: 1.25 = 1.84 (exposed) – 0.59 (control))

T/I = 60.5/1.25 = 48.4

Total dose 3 mg/m^3^ for 12 weeks;

0.003 (mg/L) × 0.03 × 60 × 12/0.03 × 7 × 12 = 181 mg/kg

IMF: 1.31 = 1.90 (exposed) – 0.59 (control))

T/I = 181/1.31 = 138

Total dose 3 mg/m^3^ for 24 weeks;

0.003 (mg/L) × 0.03 × 60 x 12/0.03 × 7 x 24 = 363 mg/kg

IMF: 1.29 = 2.11 (exposed) – 0.82 (control))

T/I = 363/1.29 = 281

Harmonic mean of T/I = 1/(1/148+1/48.4+1/138+1/281) = 105
